# Diverse genotypes of Kaposi's sarcoma associated herpesvirus (KSHV) identified in infant blood infections in African childhood-KS and HIV/AIDS endemic region

**DOI:** 10.1002/jmv.20952

**Published:** 2007-10

**Authors:** FC Kasolo, J Spinks, H Bima, M Bates, UA Gompels

**Affiliations:** 1 Virology Department, University Teaching Hospital, University of Zambia Medical SchoolLusaka, Zambia; 2 Pathogen Molecular Biology Unit, Department of Infectious Diseases, London School of Hygiene & Tropical Medicine, Keppel St., University of LondonLondon WC1E 7HT, United Kingdom

**Keywords:** Kaposi's sarcoma associated herpesvirus, HHV-8, childhood endemic KS, HIV/AIDS, virus genotype

## Abstract

Kaposi's sarcoma-associated herpesvirus (KSHV or HHV-8) has been associated with several neoplasias, including childhood endemic Kaposi's sarcoma (KS). It is possible that strain genotypes could contribute to the differences in regional presentation (mainly sub-Saharan Africa), childhood infection, lack of male sex bias, distinct disseminated forms and rapid fatality observed for childhood endemic KS. Early studies, at the advent of the HIV/AIDS epidemic, identified only the K1-A5 genotype in childhood KS biopsies as well as blood of a few HIV positive and negative febrile infants in Zambia, a highly endemic region. This current enlarged study analyses blood infections of 200 hospitalized infants (6–34 months age) with symptoms of fever as well as upper respiratory tract infection, diarrhoea, rash or rhinitis. KSHV and HIV viraemia and were prevalent in this group, 22% and 39%, respectively. Multiple markers at both variable ends of the genome (K1, K12, and K14.1/K15) were examined, showing diverse previously adult-linked genotypes (K1 A2, A5, B, C3, D, with K12 B1 and B2 plus K14.1/K15 P or M) detected in both HIV positive and negative infants, demonstrating little restriction on KSHV genotypes for infant/childhood transmission in a childhood endemic KS endemic region. This supports the interpretation that the acquisition of childhood KSHV infections and subsequent development of KS are due to additional co-factors. J. Med. Virol. 79:1555–1561, 2007. © 2007 Wiley-Liss, Inc.

## INTRODUCTION

World–wide seroepidemiological analyses of Kaposi's sarcoma associated herpesvirus (KSHV) have associated this virus with KS. Analyses of tissue biopsies by polymerase chain reaction (PCR) have identified KSHV in KS: including HIV/AIDS associated, classic (Mediterranean) and endemic (African). However, little is known about childhood endemic KS, childhood endemic KS (Sub–Saharan African regions). Childhood endemic KS can be rapid and aggressive, often presenting as lymphadenopathy, having disseminated forms. Childhood endemic KS has expanded since the HIV/ AIDS epidemic and extends to half of childhood cancers in some African countries, while practically absent in Europe or USA [Sarmati, 2004]. Is this a different strain or one now spread by HIV/AIDS as an emergent childhood disease? What are the implications for vaccine development for control? Here this is examined via analyses of genotypes in blood–borne infant infections in Zambia, a childhood endemic KS endemic region.

The highest childhood KSHV seroprevalence are in several African countries including Nigeria (20%), Uganda (21%), Zambia (47%), Egypt (44%), Tanzania (58%), and Cameroon (32%); increasing with age, for example, in adults the seroprevalence is higher in Tanzania (89%) and Cameroon (62%), as reviewed [Sarmati, 2004; Mbulaiteye et al., 2006a]. In contrast, childhood infection is rarely detected in USA and Europe (3%) [Martro et al., 2004]. Childhood infection in Africa can give rise to childhood endemic KS, but its not know whether these strains are different from adult infections, since frequent childhood infections are only observed where there is evidence for mother to child or close siblings transmission [Sarmati, 2004; Mbulaiteye et al., 2006a]. KSHV seroprevalence in African children is different fromthe relatedgammaherpesvirus,Epstein–Barr virus (EBV). By age three, 100% of children are EBV seropositive in Nigeria, and 79% of their blood DNA positive in Uganda, compared to KSHV where in these populations 20% were seropositive and 10% DNA positive in the respective studies [Martro et al., 2004; Mbulaiteye et al., 2006b]. Thus, KSHV childhood transmission is more restricted than EBV. Further, it increases with age, different from EBV. Thus, could distinct KSHV genotypes infect children compared to adults, as for Herpes simplex virus (HSV), where HSV–1 primarily infects children via saliva, while HSV–2 sexually transmitted in adults?

Previous analyses of KSHV genetic diversity by PCR on DNA extracted from adult KS biopsies followed by nucleotide sequencing, show hypervariable genes at either ends of the genome. These include K1 at the left hand end (genotypes A—F) and, K12 and K14.1/K15 (genotypes B, P, M, N, O) at the right [Cook et al., 1999; Poole et al., 1999; Zong et al., 1999; Kakoola et al., 2001]. In adult African endemic KS, two main K1 genotypes were identified A5 and B, with only a minority of the Eurasian C genotypes. While at the right hand end of the genome, there are B genotypes in K12; while there are P or M alleles for K14.1/K15, with P forms predominating in Eastern and Central Africa [Poole et al., 1999; Lacoste et al., 2000; Kakoola et al., 2001]. In contrast, in childhood endemic KS the K1–A5 genotype had been identified suggesting specific strains may infect the children (Zambia, 15 cases) [;Kasolo et al., 1998].

These diversity studies were on adult KS biopsies which have relatively high copy numbers, thus amenable to analyses. Less is known about virus genotypes infecting general populations. In adults, studies on PBMC DNA have detected K1–B and A5 genotypes in Gambia and Uganda, with similar results for adults from isolated Botswana tribes, where further variation was demonstrated [Cook et al., 1999; Meng et al., 1999; Whitby et al., 2004]. In infants, however, studies of blood DNA from early infections have only been characterized in Zambia, where previous related studies identified K1–A5 after screening conserved genes (five cases), similar to the childhood endemic KS genotype identified in this population [Kasolo et al., 1998]. In order to test whether there are restricted genotypes in infant infections in childhood endemic KS endemic regions, a further study was undertaken analyzing blood DNA from a larger group of 200 infant patients, using multiple markers for sites of variation from both ends of the genome. Both HIV positive and negative hospitalized infants were studied in this childhood KS endemic region.

## MATERIALS AND METHODS

### Patients

Peripheral blood samples were sequentially collected from 200 children, 6 months to 3 years, with non–specific febrile illness during 2003, admitted to Paediatrics Outpatients, University Teaching Hospital, Lusaka, Zambia. Fever (>38°C) was an inclusion criteria, a consistent symptom from the few studies on primary KSHV childhood infection [Kasolo et al., 1998; Andreoni et al., 2002] childhood endemic KS had not been diagnosed in this cohort. Fever arising from malaria and bacterial pneumonia were excluded and no other diagnosable cause was reported. Samples were from spent blood after routine diagnostic tests were performed. Protocols were reviewed and approved by ethical committees at the University Teaching Hospital, Zambia and London School of Hygiene & Tropical Medicine, UK.

### PCR

DNA was extracted from 200 µl whole blood (Qiagen, Crawley, UK) and resuspended in 50 µl nuclease–free H_2_O (Sigma, Gillingham, UK). PCR was carried in 20 µl reactions using 1 µl DNA with PCR master mix (Promega, Southampton, UK) and pfu polymerase with plus primers for human prolactin gene, KSHV K12 (646 bp, LGH2076/2075 and 407 bp, nested T07IF/ IR), K14.1/K15 (P,362 bp, and M,450 bpLGH2079, LGH2033, and LGH2506), HIV (BUP/UP/nested BU3) or HCMV (gN, gB1, and gB2, 150 bp) as described [Kasolo et al., 1998; Nanteza et al., 1998; Poole et al., 1999; Mattick et al., 2004; Whitby et al., 2004; Mattes et al., 2005]; plus K1 vri designed to detect all genotypes: K1vri1 5′TGTCTGCAGTCTGGCGGTTTG 3′; K1vri2 5′ACACAAGGTTTGTAAGACAGG3′ and the conserved K4 gene as control: K41 5′ TGTGGATCCAACATGGGTATCGTT 3′; K42 5′ TTTGGATCCGAGTTGAGCTGTTTAC 3′. A strict three lab policy for PCR (DNA–free lab reagent addition, DNA lab for DNA addition, PCR lab for amplifications) was used against contamination and none detected; water negative controls for every 5–10 reactions, were negative. KSHV sequences collected had unique signatures.

### Nucleotide Sequencing and Analysis

The amplified PCR products were purified (Qiagen MinElute), followed by nucleotide sequencing using BigDye v3.1 (ABI) and ABI 3730 analyzer as described [Mattick et al., 2004]. Sequence analyses used Chromas with Genbank comparisons using Blast and multiple alignments with ClustalW [Chenna et al., 2003]. Phylogenetic trees were constructed using Phylip programs, with Blosum62 distance matrix, a topological tree algorithm and 100 bootstrap analyses [Brodsky et al., 1992; Felsenstein, 2004]. Sequence submitted to EBI/Genbank.

## RESULTS

### Detection of KSHV

DNA was extracted from peripheral whole blood samples from the cohort of 200 febrile infants, hospitalized in Lusaka, Zambia. These were then analyzed by PCR and sequencing using primers specific for KSHV as well as human DNA control (Prolactin). All KSHV sequences collected had unique signatures and there was no evidence for PCR contamination. Moreover, many sequences subsequently derived had not been previously identified in the laboratory. One hundred and forty–one samples were included in the analyses which showed consistent results, and a positive human prolactin gene control.

PCR detection of HIV and KSHV was also compared with human cytomegalovirus (HCMV), another herpesvirus related to morbidity and mortality in these HIV positive children. The results showed HIV, KSHV, and HCMV detected in 39%, 22%, and 11% infants, respectively (Table I). KSHV and HCMV coinfections were rare (3/14 HCMV positive). Although co–infections with HIV were more frequently identified with KSHV than with HCMV (65% vs. 47%) supporting the expansion of this infection with HIV/AIDS consistent with studies in adults.

**TABLE 1:** KSHV Genotypes in Hospitalized Febrile Infants (6–34 months) in Zambia

Patient^a^	HIV	HCMV	KSHV	K1	K12	K14.1/15	Age	Symptoms
K2	+	−	+	A5	B1	P	6 mo	URTI
K3	−	−	+	A5	B1	P	8 mo	URTI
K4	+	−	+	A5	B2	P	19 mo	URTI, Rash
K33	−	+	−	—	—	—	6 mo	URTI
K35	+	−	+	un	B1	P	11 mo	URTI, Diarrhoea
K37	+	−	+	un	B2	P	15 mo	Fever
K42	−	−	+	un	B2	P	11 mo	Fever
K46	+	−	+	B	B1	un	7 mo	Fever, Rhinitis
K50	+	−	+	A2	B2	P	10 mo	URTI
K54	+	+	+	A2	B1	P	24 mo	Fever
K55	+	−	+	un	B1	P	29 mo	Fever
K57	+	+	−	—	—	—	17 mo	Fever, Diarrhoea
K58	+	−	+	A2	B1	P	3 yr	Fever
K60	−	+	−	—	—	—	14 mo	Fever
K61	+	+	−	—	—	—	14 mo	Fever, Rash
K73	+	−	+	A2	B2	P	13 mo	URTI
K74	+	−	+	A5	B2	P	11 mo	Fever
K75	+	−	+	un	B2	un	12 mo	Fever
K78	−	−	+	un	B1	P	15 mo	URTI
K79	+	−	+	B	B1	P	19 mo	Fever
K86	+	+	−	—	—	—	6 mo	Fever
K91	+	−	+	un	B1	P	10 mo	URTI, Diarrhoea
K93	+	+	+	un	B2	un	22 mo	Fever, Diarrhoea
K111	−	−	+	ABCD	B1	un	9 mo	Fever
K113	−	−	+	B	un	un	11 mo	Fever
K117	+	−	+	B	B1	P	16 mo	URTI
K118	+	−	+	C3	un	M	27 mo	Fever, Diarrhoea
K135	+	+	−	—	—	—	20 mo	Fever
K136	+	−	+	A5	B2	P	23 mo	URTI
K137	−	+	+	A3	un	un	6 mo	Fever
K140	−	−	+	C3	un	un	8 mo	Fever
K141	−	+	−	—	—	—	19 mo	URTI, Diarrhoea
K149	+	−	+	C3	un	un	30 mo	Fever, Diarrhoea
K162	−	−	+	C3	un	un	11 mo	Fever
163	−	−	+	un	B1	un	34 mo	Fever
K164	−	−	+	C3	un	un	14 mo	URTI
K166	−	+	−	—	—	—	17 mo	URTI
K180	−	+	−	—	—	—	9 mo	Fever
K186	−	−	+	C3	un	un	11 mo	Fever
K190	−	+	−	—	—	—	21 mo	URTI and Rash
K192	+	+	−	—	—	—	18 mo	Fever and Rash
K199	−	−	+	C3	un	un	9 mo	Fever

un, KSHV DNA+,but undetectable by primers indicated; mo,months age;yr, year.

a Patients positive for KSHV or HCMV are shown URTI, upper respiratory tract infection [Fever with Rhinitis or Pharyngitis or tonsillitis].

### Clinical Features KSHV Infected Infants

The PCR reaction (1/50 of 0.2 ml blood with sensitivity of 10–100 copies) detected at least 2,500 copies/ml blood, consistent with KSHV viremia. Given the ages of the patients (6–34 months), these are most likely primary infections, with possible maternal or sibling transmission through non–sexual routes as suggested previously for this population and neighboring regions [He et al., 1998; Mbulaiteye et al., 2003, 2006a; Dedicoat et al., 2004]. Fever was the inclusion criteria and other hospital diagnosed causes of fever were excluded (malaria or bacterial infections). Amongst the 31 infants who were PCR positive for KSHV, upper respiratory tract infections were the most common symptom 45% (14/31), followed by diarrhea 19% (6/31), rash 3% (1/31) and rhinitis 3% (1/31) (Table I).

### Nucleotide Sequencing and Genotype Analyses

**Fig. 1:**
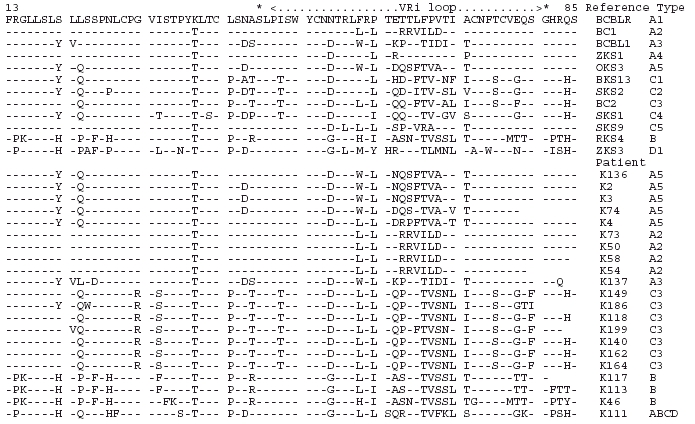
Sequence analyses of K1 variable region, VRI loop, identifies genotype diversity in childhood KSHV from African endemic region. The K1 region was PCR amplified from blood DNA, sequenced and aligned against published representatives of K1 genotypes [Zong et al., 1999]. Dashes indicated identity.

**Fig. 2:**
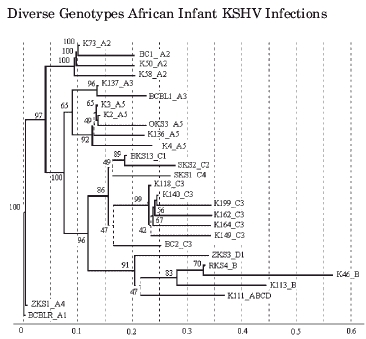
Phylogenetic tree of K1 sequences from childhood KSHV blood infections in comparison to reference genotypes from Figure 1 shows extensive diversity. Scale and branch lengths indicate relative genetic distance. In the bootstrap analyses the multiple alignment was resampled generating 100 trees. Bootstrap values achieved are expressed in percentages and placed at the nodes.

Many samples were at the limit of detection by PCR, which restricted subsequent multiple analyses by nucleotide sequencing. The PCR positive K1 loci was first examined by sequencing. The results showed the K1 A5 genotype, previously identified in infected children and in childhood KS in this region, but also unexpected diversity. The K1 A5 genotype was common 23% (5/22), now linked here with K12 B1 or B2 and K14.1 P genotypes, consistent with previous results for a distinct strain in this region (Table I and Fig. 1), and presenting with respiratory symptoms (4/5). However, also shown were K1–B (4/22), (prevalent in African adult KS) as well as K1–A2, A3, and C3, previously common in adult Mediterranean/Asian/Europe KS (C3, 7/22). There were HIV positive and negative examples of all. The Zambian infant blood KSHV sequences clustered with representative of K1 A, B, C, or D genotypes from adult KS biopsy samples (Fig. 2).

**Fig. 3:**
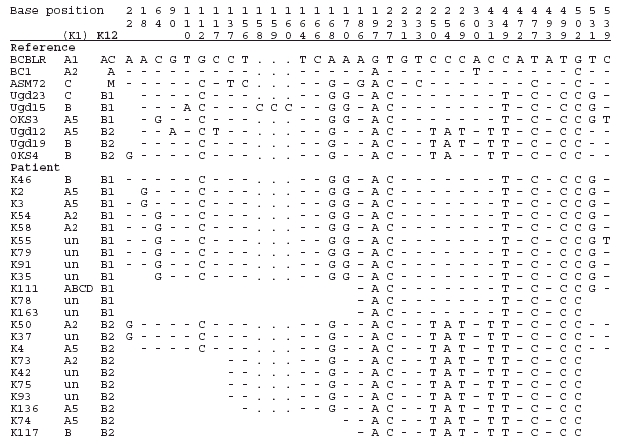
Nucleotide changes in the K12 region shows B genotypes identified in African regions. Reference genotypes are shown (A, AC, M, B1, and B2), including Ugandan reference sequences from adultKS biopsy materials, compared to the Zambian infant patients blood genotypes showing B1 and B2. The K12 segment is in the reverse complement orientation (coding) from the genomic sequence. The positions of nucleotide variation and reference sequences are as shown previously with position 1 as in [Poole et al., 1999; Kakoola et al., 2001] or 118,065 in the BC1 genomic sequence, or position 51 in Poole et al. [1999], (positions −22 is 30, and 64 is 115 in Poole et al. [1999];). K12ORF, Kaposin A, ends at position 147 as inPoole et al. [1999] and Kakoola et al. [2001] or 117,919 in the BC1 genome [Russo et al., 1996]. The PCR product was from 118,116–117, 469. Hyphens and dots indicate identical or deleted residues.

At the loci from the right hand of the genome K12 and K14.1/K15 genotypes were identified, using primers to amplify regions as characterized previously [Poole et al., 1999]. Previous studies showed divergence of nucleotide sequences of K12 at selected positions, mostly intergenic non–coding, and some used as representative genotypes (Fig. 3). There were 21 Zambian infant K12 genotypes identified and these clustered with K12 B1 and B2 genotypes, similar to sequences identified in adult KS biopsies in other parts of Africa as shown [Poole et al., 1999; Kakoola et al., 20017rsqb; (Fig. 3). The PCR based assay for the K14.1/15 distinguishes by size the P or M genotypes. The dominance of the K14.1/15 P genotype (17/18) is in agreement with previous studies showing predominance in Eastern African regions also in adult KS biopisies (Table I) [Lacoste et al., 2000]. For all sites analyzed some samples could not be detected by the primers used, suggesting further diversity.

One K1 sequence overlapped all of the genotypes, ABCD (K111), either a local progenitor virus or effects of recombination (Figs. 1 and 2). This was distinct from previous progenitor strains (K1–43Berr closest, 18% divergence) [Lacoste et al., 2000; Whitby et al., 2004]. However, the K12 B1 genotype from this sample was similar to other B1 sequences from the region.

## DISCUSSION

In earlier studies, of a childhood endemic KS region, Zambia, analyses of blood infection in five infants showed only K1–A5 genotypes and this correlated with the genotype identified in different infant and childhood KS samples [Kasolo et al., 1998]. Here the origin of the KSHV strains infecting infants is analyzed in a larger study of blood infections in 200 hospitalized febrile infants. KSHV sequences were identified in 31 individuals. Given the young age (6–34 months) of the patients, and the relative levels detected, these appeared to be primary or early infections of which little is known. In addition to fever, many had symptoms of upper respiratory tract infections or diarrhoea, but no evidence yet for KS. Multiple loci were used to genotype the virus. The results showed diverse adult–like genotypes identified in both HIV positive or negative infants. These included the following K1/K12/K14.1/15 genotypes: K1–A5, A2, A3, B, C3 or A/B/C/D; K12–B1 or B2; K14.1/15 P or M. It is also possible that given rapid increases in HIV/AIDS in Zambia, the genotype pool of KSHV may have expanded, leading to emergence of diverse childhood infections. Recently, blood DNA from five older children (>5 years age) in Uganda also showed 3 K1–A5 and 2 K1–B [Mbulaiteye et al., 2006a]. This is consistent with results shown here, although here further loci have been analyzed, with more diversity identified in the Zambian region in a larger group of younger children and infants. Thus, in a childhood KS endemic region, childhood KSHV infection can occur with diverse genotypes.

**Fig. 4:**
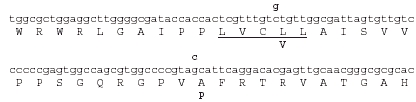
Coding changes in the K12 Kaposin A gene. Zambian infant blood KSHV K12 B genotypes (from Fig. 3) compared to protein encoded by reference P type genome (GK18) [Rezaee et al., 2006]. A disrupted motif reported required for transformation is underlined [Tomkowicz et al., 2005].

Despite the diversity of genotypes identified in the infant blood KSHV infections, is there any evidence for distinct strains for childhood transmission? The K1A5 genotype is prevalent in KS biopsy samples from patients in regions of Africa where there is childhood endemic KS [Kasolo et al., 1998; Lacoste et al., 2000; Kakoola et al., 2001]. Furthermore, the P genotype of K14.1/15 is also linked to this region [Lacoste et al., 2000], where only one M genotype was identified. Moreover, only the K12 B genotype (both B1 and B2) was identified in these infant infections, and these genotypes are dominant in adult KS identified in other African regions [Poole et al., 1999; Lacoste et al., 2000; Kakoola et al., 2001]. Most of the K12 variation is in the 30 non–coding intergenic region. However, the encoded Kaposin A is a tumorigenic transforming gene interacting with cytohesin–1 integrin regulator, and two coding changes were identified (Fig. 4) [Muralidhar et al., 1998; Kliche et al., 2001]. One shown in some B1 genotypes,C64G gives rise to Leu34 to Val, though conservative, it disrupts an LXXLL motif (to LXXVL), reported required for cellular transformation [Tomkowicz et al., 2005]. The other substitution is divergent, G112C, giving Ala50 to Pro in all B genotypes compared to both referenceMand P type genomes (BC1 and GK18, respectively) [Russo et al., 1996; Rezaee et al., 2006]. Thus several genotype markers may link with childhood endemic KS endemic regions. However, the observation of multiple K1 genotypes and past evidence for linked genome diversity [Poole et al., 1999], suggests multiple KSHV strains can be transmitted to infants.

Since the data shown here indicates there does not appear to be any strain restriction for infant infection in a region endemic for childhood KS, this implicates other cofactors for childhood KSHV transmission and KS development. Seroepidemiological studies on KSHV transmission, identify factors including genetic predisposition, low socioeconomic status, immunosuppresion from HIV/AIDS or other influences, plus surface water exposure, possibly linking to hygiene [DeSantis et al., 2002; Mbulaiteye et al., 2003, 2005; Plancoulaine et al., 2003; Klaskala et al., 2005]. In this regard, evidence for mother to child or close siblings transmission in African KS endemic populations has been demonstrated [He et al., 1998; Mbulaiteye et al., 2003, 2006a; Dedicoat et al., 2004] with virus detected in saliva and urine [Beyari et al., 2004; Brayfield et al., 2004; Mbulaiteye et al., 2004]. Factors for development of adult KS require KSHV seropositivity, and include linkage to higher socioeconomic status, water exposure, HIV/ AIDS immunosuppression, or immunogenetic regulation [Ziegler et al., 1997, 2003; Sitas and Newton, 2001; Gaya et al., 2004]. However, not all African countries with high childhood KSHV seroprevalence have high childhood endemic KS, thus additional factors affect childhood endemic KS development. Whether these African childhood infections with diverse KSHV genotypes as shown here, have differences in subsequent KSHV transmission or KS development, remains to be determined and has important implications for vaccine design.
